# Integrating environmental, molecular, and morphological data to unravel an ice-age radiation of arctic-alpine *Campanula* in western North America

**DOI:** 10.1002/ece3.1168

**Published:** 2014-09-26

**Authors:** Eric G DeChaine, Barry M Wendling, Brenna R Forester

**Affiliations:** 1 Department of Biology, Western Washington University516 High St., Bellingham, Washington, 98225; 2 University Program in Ecology, Nicholas School of the Environment, Duke UniversityBox 90328, Durham, North Carolina, 27708

**Keywords:** Arctic-alpine plants, ecological niche modeling, morphology, Pacific Northwest, Quaternary, statistical phylogeography

## Abstract

Many arctic-alpine plant genera have undergone speciation during the Quaternary. The bases for these radiations have been ascribed to geographic isolation, abiotic and biotic differences between populations, and/or hybridization and polyploidization. The Cordilleran *Campanula* L. (Campanulaceae Juss.), a monophyletic clade of mostly endemic arctic-alpine taxa from western North America, experienced a recent and rapid radiation. We set out to unravel the factors that likely influenced speciation in this group. To do so, we integrated environmental, genetic, and morphological datasets, tested biogeographic hypotheses, and analyzed the potential consequences of the various factors on the evolutionary history of the clade. We created paleodistribution models to identify potential Pleistocene refugia for the clade and estimated niche space for individual taxa using geographic and climatic data. Using 11 nuclear loci, we reconstructed a species tree and tested biogeographic hypotheses derived from the paleodistribution models. Finally, we tested 28 morphological characters, including floral, vegetative, and seed characteristics, for their capacity to differentiate taxa. Our results show that the combined effect of Quaternary climatic variation, isolation among differing environments in the mountains in western North America, and biotic factors influencing floral morphology contributed to speciation in this group during the mid-Pleistocene. Furthermore, our biogeographic analyses uncovered asynchronous consequences of interglacial and glacial periods for the timing of refugial isolation within the southern and northwestern mountains, respectively. These findings have broad implications for understanding the processes promoting speciation in arctic-alpine plants and the rise of numerous endemic taxa across the region.

## Introduction

The dynamic interplay between the climatic variability of the glacial cycles and the rugged topography of mountain landscapes provided many opportunities for the diversification and speciation of arctic-alpine plants during the Quaternary (Abbott et al. 2000). Whether or not the Pleistocene promoted speciation is under debate (Haffer 1969; Avise et al. 1998; Schoville et al. 2012). In the case of arctic-alpine plants, it is clear that speciation did occur and is ongoing today. Indeed, the Arctic is no longer thought of as an evolutionary “freezer” as posited by some (e.g., Allen et al. 2006), but rather the flora is known for a relatively high number of Pleistocene speciation events (reviewed in Brochmann and Brysting 2008).

The main modes of genetic divergence and speciation in plants were heightened for the arctic-alpine flora during the Quaternary. First, isolation over many generations, whether between glacial refugia or interglacial sky islands, promoted genetic differentiation through genetic drift. For some taxa, new species arose simply through isolation in the absence of ecological differentiation (Kadereit et al. 2004; Valente et al. 2010). That said the vast majority of studies to date have highlighted the importance of remixing, hybridization, and polyploidization following isolation in the process of recent arctic-alpine plant speciation (Brochmann et al. 2004). Finally, environmental conditions and biotic communities likely varied among refugia, and the different selective pressures imposed by those environments provided the opportunity for ecological speciation (reviewed in Givnish 2010). This includes not only abiotic, but also biotic factors – variation among the available pollinators would have been of particular importance to plant reproduction, divergence, and floral evolution (Grant 1949; Nakazato et al. 2013). The aforementioned factors could have acted independently or in concert to promote genetic divergence.

The western region of North America is geographically complex with a high degree of environmental heterogeneity, affording plants a number of unique habitat types and biotic interactions. Accordingly, 60% of the vascular plant genera endemic to North America are limited to the western portion of the continent (Qian et al. 2007). During glacial episodes, massive sheets of ice grew over much of the continent, and in conjunction with the expansion of mountain glaciers, isolated regions and their inhabitants. Several potential glacial refugia have been identified in the west, including Beringia, Haida Gwaii, Vancouver Island, the Olympic Peninsula, the Southern Cascades (including the Columbia Gorge and the Klamath region), and the Northern and Southern Rocky Mountains (Hultén 1937; Soltis et al. 1997; Swenson and Howard 2005; Shafer et al. 2010). Furthermore, the north–south running cordillera provided opportunities for the persistence of arctic-alpine plants during cold and warm climatic periods (Allen et al. 2012; DeChaine et al. 2013a,b; Marr et al. 2013). In general, during interglacials, populations expanded from refugia, but along a chain of sky islands that reduced the connectedness among alpine populations and potentially led to local extinction through habitat reduction. Thus, isolation among populations likely impacted the evolution and diversification of arctic-alpine taxa during both glacial and interglacial stages. Additionally, environmental conditions, resources, communities, and biotic interactions have varied along the length of the cordillera. The high proportion of geographically limited species of mountain plants in western North America is likely a product of the interplay between climate and topography and the associated cycles of isolation and admixture among neighboring populations (DeChaine et al. 2013a,b).

The arctic-alpine *Campanula* L. (Campanulaceae Juss.) of western North America present an excellent opportunity for investigating the underlying factors that have promoted speciation and given rise to the high proportion of endemics across the region. Members of this genus have undergone at least three episodes of diversification following colonization of the continent (Morin 1983; Haberle et al. 2009; Wendling et al. 2011). The Cordilleran *Campanula*, comprised of approximately seven mostly endemic species, experienced a rapid radiation after colonization of North America in the Pleistocene (Wendling et al. 2011). Members of this clade are patchily distributed along the fragmented mountain landscape of the west, ranging from the Brooks Range of Alaska (and eastern Siberia) southwards along the Coast, Cascade, Olympic, and Rocky Mountain ranges to northern California and southern Colorado. In addition, several other genera within the Campanulaceae, including *Triodanis* Raf. ex Greene and the *Campanula-Githopsis* Nutt.*-Heterocodon* Nutt. clade, have diversified in North America (Morin 1983). The reoccurring theme of colonization, diversification, and endemism begs the question as to what ecological and evolutionary forces have brought about this pattern. Geographic isolation has been cited as the basis for genetic divergence and endemism in some *Campanula* clades (Cellinese et al. 2009; Haberle et al. 2009). Exposure to different environmental conditions has also likely played a role in speciation within the family. In addition to abiotic factors, floral morphological diversity in *Campanula* has been driven in large part by pollinator-mediated selection on floral traits (Roquet et al. 2008; Scheepens et al. 2011), and this could be a factor in the diversification within the Cordilleran *Campanula* as well. It is important to note that the various factors promoting genetic divergence and speciation are not mutually exclusive, but are potentially all pieces of the evolutionary puzzle for the radiation of some arctic-alpine *Campanula* clades during the Quaternary.

**Figure 1:**
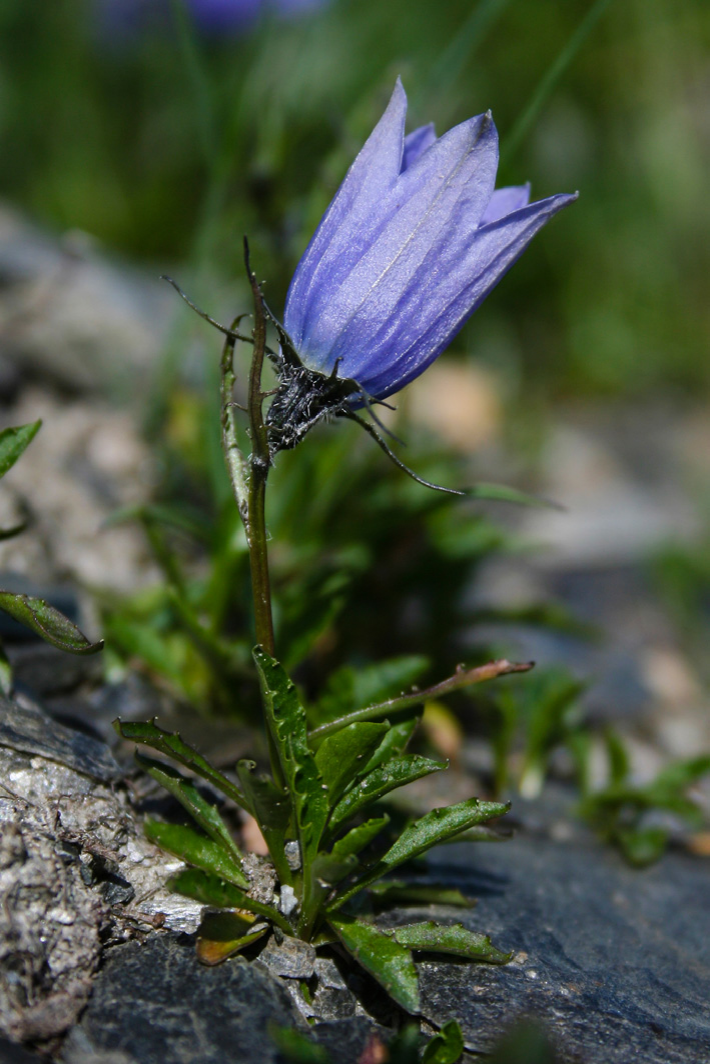
A representative of the Cordilleran *Campanula*: *C. lasiocarpa* in arctic Alaska. Photo credit EG DeChaine.

The objective of our study was to investigate the factors that promoted the recent and rapid radiation of the Cordilleran *Campanula* (see Fig.1 for an image of *C. lasiocarpa* Cham., a member of this clade). Specifically, we asked what stimulated speciation in this clade. As such, our study was focused at the species level, and we did not investigate the infraspecific (e.g., population genetic) diversity within each taxon. To accomplish this goal, we took an integrated approach to examining the speciational history of this group. First, we created paleodistribution ecological niche models (ENMs) and identified potential Pleistocene refugia (regions that likely harbored suitable habitat throughout the Pleistocene) for the clade, which were then used to generate biogeographic hypotheses [(1) multiple refugia, (2) northward expansion from a southern refugium, and (3) southward expansion from a northern refugium] based on potential scenarios of refugial isolation and dispersal. We estimated the phylogenetic history of the Cordilleran Clade by reconstructing a species tree and divergence times among taxa using eleven noncoding nuclear loci and used these inferences to test the biogeographic hypotheses in a maximum-likelihood framework. Additionally, geographic and climatic data were used to calculate niche overlap, equivalency, and similarity for individual taxa. The niches of sister taxa (as inferred in the phylogeny) were compared to test the hypothesis that varying environmental pressures contributed to speciation. Finally, we assessed the differences among the Cordilleran taxa based on twenty-eight morphological characters to test the hypothesis that differing selective pressures imposed by pollinators affected speciation. As with the niche overlap analysis, morphological variation between sister taxa was assessed. Clearly, these three hypotheses (biogeography, abiotic environment, and pollinator community) are not mutually exclusive, and the relative importance of each process potentially varied among the divergence events within the clade. Because of this, we investigated the relative importance of each potential causal factor on divergence by comparing speciation events across the clade. This integrated approach provided a means for synthesizing diverse datasets to better understand the evolutionary history of arctic-alpine plants and the factors that have given rise to endemic taxa in mountain environments.

## Materials and Methods

### Specimen sampling

**Figure 2:**
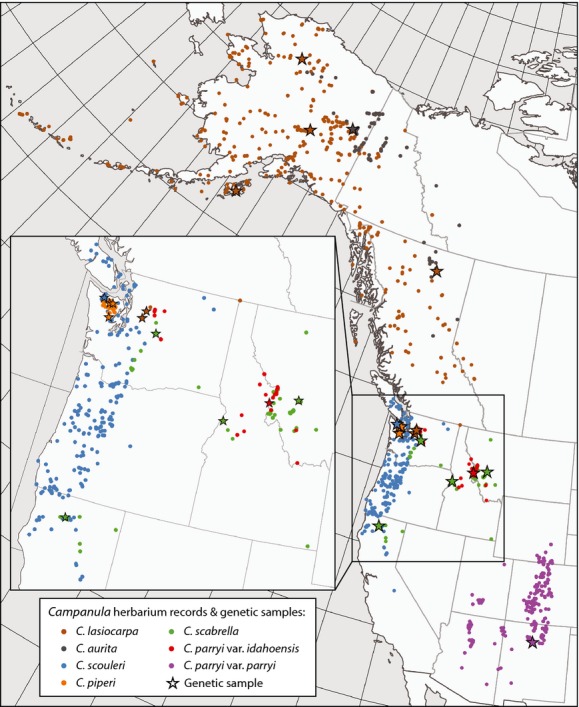
Distribution of herbarium records (points) and genetic samples (stars) for the Cordilleran *Campanula* in western North America.

All analyses of the Cordilleran *Campanula* (Wendling et al. 2011) were performed at the species level. We sampled species from across their range (Fig.2, Tables S1, S2), with sample sizes for the molecular analyses in parentheses: *C. aurita* Greene (*n* = 1), *C. lasiocarpa* (*n* = 7)*, C. parryi* var*. idahoensis* McVaugh (*n* = 1)*, C. parryi* var*. parryi* A. Gray (*n* = 1)*, C. piperi* Howell (*n* = 4)*, C. scabrella* Engelm. (*n* = 4)*,* and *C. scouleri* Hook. ex A. DC (*n* = 1). Though likely a member of this clade (Mansion et al. 2012), samples of *C. shetleri* Heckard were not available for this study. We used a specimen of *C. rotundifolia* L. as an outgroup in the molecular analyses. Individuals were either collected in situ or acquired on loan from herbaria. Freshly collected individuals were desiccated on silica gel, and voucher specimens were archived in the Pacific Northwest Herbarium (WWB).

### Paleodistribution modeling

Ecological niche models were built for the combined Cordilleran *Campanula* using georeferenced herbarium records (Fig.2) and CRU TS 2.1 climate data for the period 1971–2000 at 0.5° resolution (Mitchell and Jones 2005). One record was used per 0.5° pixel, yielding a combined sample size of 409. The *Campanula* were modeled as a clade to be as generous as possible in assessing suitable refugia for the development of phylogeographic hypotheses. Predictor variables were selected using the random forest (RF) algorithm (Cutler et al. 2007; Strobl et al. 2008) in R v. 2.13.1 (R Development Core Team 2012). Five variables were selected from an initial group of 44, based on RF output and Spearman's correlation values (variables correlated at rho values ≤ |0.7|): annual temperature range (maximum temperature of the warmest month minus minimum temperature of the coldest month); mean diurnal range for summer (mean of the maximum temperature minus minimum temperature of each month for June, July, August); summer minimum temperature (mean of minimum temperatures for June, July, August); and precipitation of the wettest and driest months.

An ensemble modeling approach was used to model species distributions, following Forester et al. (2013). Briefly, modeling was conducted with the BIOMOD package (Thuiller et al. 2009) in R and Maxent, v. 3.3.3 (Phillips et al. 2006). Eight model algorithms were run: generalized linear models, generalized additive models, multivariate adaptive regression splines, classification tree analysis, flexible discriminant analysis, generalized boosted models, random forest, and maximum entropy. For all models, the entire background of the study area was used to create background data, with these data weighted to maintain the number of presence records equal to the number of background records.

Model performance was assessed using ten bootstrapped replicates (70% training, 30% verification) for each algorithm. The area under the curve of the receiver operating characteristic (AUC, Fielding and Bell 1997) was averaged across replicates to determine whether models should be removed from the ensemble (AUC < 0.7). Final models were built using 100% of the available data (Araújo et al. 2005). Model probabilities were converted to presence/absence predictions using two thresholds: minimizing the absolute value of sensitivity minus specificity and the mean probability value across model output (Liu et al. 2005).

To create paleodistributions, models were projected onto climate data from the mid-Holocene (6 KYA), Last Glacial Maximum (LGM; 21 KYA), and Last Interglacial (LIG; 124 KYA). Paleoclimate data were interpolated to 0.5° resolution using ordinary cokriging in ArcMap (ESRI 2010). Mid-Holocene and LGM climate data were derived from three general circulation models (GCMs): CCSM 3 (Otto-Bliesner et al. 2006), HadCM3 (Gordon et al. 2000), and MIROC 3.2 (K-1 Model Developers 2004). Data for the LIG were provided through the HadCM3 model. Paleodistributions for the mid-Holocene and LGM represent consensus across these three GCMs. Maps for each time period represent the percentage of models voting “suitable habitat” for a given pixel based on 16 (current and LIG) or 48 (mid-Holocene and LGM) model votes. The final refugial map was derived by assessing the probability of suitable habitat (50% and 75%) in each pixel across all four time slices. The LGM ice layer (Dyke et al. 2003) was overlaid to exclude potential suitable habitat under the ice sheet and create the final map of potential refugia.

### Molecular analyses

We generated a multi-locus nuclear DNA (nDNA) dataset of anonymous noncoding markers for estimating the evolutionary history of *Campanula* of interest. Our approach to designing the anonymous locus primer sets (ALPS) for plants followed DeChaine et al. (2013b), using Invitrogen's (Carlsbad, CA) TOPO® Shotgun Subcloning Kit on genomic DNA extracted from leaf tissue of *C. lasiocarpa* (accession WWB 22731) using Qiagen's (Valencia, CA) DNeasy® Plant Extraction kit. To select the appropriate loci for use as ALPS, we ran blastn and blastx searches against the nucleotide databases of the National Center for Biotechnology Information. We discarded DNA fragments matching organelle or protein coding sequences and removed sequences that had long open reading frames. Fifteen sequences were selected for designing ALPS, and primer pairs were optimized in Primer3-web 0.4.0 (Rozen and Skaletsky 2000). Ultimately, 10 ALPS reliably produced clean PCR products and were chosen for sequencing (Table1; see Table S3 for taxon/locus matrix). We included the internal transcribed spacer (ITS) region as an eleventh locus following rigorous quality control (Wendling et al. 2011).

**Table 1:** Locus-specific substitution models and statistics.

Locus	*N*	bp	Indel	Primers	Model	Rate	*N*_h_	*h*	*θ*_S_	*π*	*D*	D*	F*
ALP 03	20	897	8	TCGACACAACTCTTTTCATTGC	TMI2 + I	1.00	19	0.995	0.124	0.123	−0.046	0.152	0.107
				GGTCGGAAATCATACCATAACC									
ALP 04	19	889	4	GGTCCTGGCATCTAATTTGC	HKY	0.24	19	1.000	0.315	0.018	−1.699*	−1.598*	−1.900*
				CTGATCATCACCGACAATCG									
ALP 06	18	756	2	TCTTGAGTGAAAGATCCCATCC	HKY	0.12	17	0.993	0.031	0.014	−2.253**	−2.978**	−3.212**
				GCAAAGGAGACTTACGAAACG									
ALP 13	16	932	0	CATCAATCCAACAACGTTCC	TIM1 + G	1.09	16	1.000	0.104	0.097	−0.263	−0.275	−0.314
				ATCCCCCATTGTTGTGTAGC									
ALP 14	23	*467*	2	ACCACTGTGGGGTTTTATGG	TPM2uf + I + G	0.80	22	0.996	0.113	0.075	−1.328	−1.741	−1.892
		255		GGAAGAGAGATGTTCTGCTTGG									
ALP 19	26	*966*	3	CGTTCACTACCCCTTCATGG	TVM + I + G	0.69	26	1.000	0.128	0.060	−2.079**	−2.686**	−2.934**
		599		TGGTTCATCTCCCACTAGACC									
ALP 24	13	*768*	0	CATAAGCGGACTCTAGCAAGC	TPM2uf + I + G	0.33	13	1.000	0.079	0.054	−1.307	−1.885	−1.996
		730		GCGTTCTTGGTCTTTGAAGG									
ALP 25	13	519	0	TGTGTGGCTCACATTCATGG	TIM2 + I + G	0.27	12	0.994	0.048	0.038	−0.873	−1.452	−1.490
				TAGGCGGATCTTCCAATACG									
ALP 31	19	774	0	CTTCATTCAAGCCCAAGACC	GTR + I + G	0.24	18	0.994	0.061	0.050	−0.704	−0.718	−.0841
				GAAAGGTTGGGGTATGTTGG									
ALP 32	14	847	2	GCGATTTGAAGGTGAAGACC	GTR + G	1.53	14	1.000	0.190	0.126	−1.528	−1.516	−1.747
				CATCAACACCCCAATCTCG									

The number of sequences (*N*) used in this study, the length of the locus (bp) pre- (italics) and post (regular)-trimming as determined by recombination in RDP3, the number of indels, and the forward and reverse primers are given. The model of DNA substitution from JMODELTEST (Model), and the relative interlocus substitution rate from *BEAST (Rate) are shown. From DNASP, the number of haplotypes (*N*_h_), haplotype diversity (*h*), the per site measure of theta based on segregating sites (*θ*_S_) and nucleotide diversity (*π*) and test of neutrality through Tajima's *D*, and Fu and Li's D* and F* are shown where *P < 0.1 and **P < 0.05.

Anonymous locus primer sets (ALPS) were PCR amplified (following the protocol of DeChaine et al. 2013b), cloned using TOPO® TA cloning kits, and three clones per sample (when possible) were sequenced in both directions by the University of Washington's High-Throughput Genomics Service Unit. In preparation for the analyses, all genetic datasets were edited on SEQUENCHER v4.8 (GeneCodes Corp., Ann Arbor, MI), aligned using CLUSTALX 2.0 (Larkin et al. 2007), and alignments were manually edited in MACCLADE 4.08a (Maddison and Maddison 2000). We coded indels (most of which separated the Cordilleran *Campanula* from *C. rotundifolia*) as simple binary characters (Simmons and Ochoterena 2000) and excluded all sites of ambiguous alignment from further analyses. We tested each locus for recombination with the RDP (Martin and Rybick 2000), GENECONV (Padidam et al. 1999), MAXCHI, and CHIMAERA (Posada and Crandall 2001) algorithms implemented in the software package RDP3 (Martin and Rybick 2000). These approaches were chosen because the first two detection methods identify recombinants as regions where the percent similarity is higher than others, while the latter two use the proportion of variable and nonvariable sites to identify breakpoints. Recombinant individuals and/or segments that were detected by >1 detection method were excluded from the study: Approximately 5% of all sequences were discarded, and ALPS 14, 19, and 24 were trimmed by 212, 367, and 38 bp, respectively.

Using DNASP v. 5 (Librado and Rozas 2009), we calculated haplotype diversity (*h*), the number of segregating sites (*θ*_S_), and nucleotide diversity (*π*), and performed neutrality tests for each ALP by comparing Tajima's D (Tajima 1989) and Fu and Li's D* and F* (Fu and Li 1993) statistics to 1000 coalescent simulations of a large, neutrally evolving population.

### Testing biogeographic hypotheses

The relationships among the *Campanula* were inferred in BEAST 1.6.2 (Drummond and Rambaut 2007), using the *BEAST (Heled and Drummond 2010) method of phylogeny estimation, applying the Yule tree prior, and allowing rates to vary among loci. This approach avoids problems associated with estimating trees from concatenated loci (Edwards et al. 2007; Heled and Drummond 2010). Because the ALPS were designed for the in-group, we were not able to sequence all loci for all individuals. Missing loci were coded as missing data so that they would not bias tree reconstruction and divergence time estimates (Wiens and Morrill 2011). To evaluate the sensitivity of our analyses to the small and variable sample sizes per taxon (including *n* = 1), we performed the analyses using three separate population size models: (1) constant, (2) linear, and (3) linear and constant root. Because tree topologies and branch lengths did not differ among the models (data not shown), we assumed our sample sizes did not bias phylogenetic reconstruction. The analyses were run for one billion generations with 90% of each run discarded as burn-in. Stationarity was determined by examining parameter trend plots and effective sample size (ESS) values (all > 200) in the program TRACER 1.5 (Rambaut and Drummond 2007). All analyses were repeated three times using different random seeds to confirm convergence of parameter estimates. To calculate divergence dates, we used a range from (1) the general *μ* for plant autosomal genes of 7.1 × 10^−9^ ± 0.7 substitutions/site/generation (Ossowski et al. 2010) that fits with an earlier estimate of 7.0 × 10^−9^ ± 1.2 (Wolfe et al. 1987) to (2) 1.5 × 10^−8^ ± 0.5, determined by Koch et al. (2000), which may be more appropriate given that our loci are all noncoding. The Koch et al. (2000) estimate is similar to the rate for noncoding regions inferred by Ossowski et al. (2010). Our best estimate of reproduction in the Cordilleran Clade relies on the closely related and geographically and environmentally similar perennial, *C. rotundifolia*, which does not flower in its first year (Nuortila et al. 2004). Following this, and given the short growing season in arctic-alpine environments, we assumed a generation time of 2 years.

Next, we used LAGRANGE v. 20130526 (Ree and Smith 2008), a maximum-likelihood framework for testing models of geographic range dynamics based on a dispersal–extinction–cladogenesis model, to test biogeographic hypotheses. The three biogeographic hypotheses based on the paleodistribution models were as follows: (1) multiple refugia (symmetric dispersal at a rate of 1.0 to and from adjacent areas), (2) northward expansion (rate of 1.0 northward dispersal from southern refugia and 0.001 southwards), and (3) southward expansion (rate of 1.0 southward dispersal from northern refugia and 0.001 northwards). Each of the models was temporally stratified over two time periods: 1100 KY (because the faster *μ* = 1.5 × 10^−8^ estimated the root at 1030 KYA) and 2250 KY (because *μ* = 7.1 × 10^−9^ estimated the root at 2183 KYA) with potential dispersal differing between glacial and interglacial periods. The temporal chronology for glacial and interglacial periods was based on the Wisconsinan, Illinoian, and PreIllinoian in North America and the correlated MIS stages (Richmond and Fullerton 1986; Winograd et al. 1997; Gibbard et al. 2007). The phylogeny and range matrix defining the region from which each species was collected were uploaded to the LAGRANGE CONFIGURATOR (Ree and Smith 2009) allowing for two potential ancestral regions per node, and a python file was configured for use in the LAGRANGE analyses.

One limitation of this approach is that the time frame for most speciation events inferred in the phylogeny is older than the paleodistribution models used to create the biogeographic hypotheses. We used the potential geographic distributions during LIG, LGM, and present to estimate where refugia (defined here as the availability of suitable habitat during both glacial and interglacial times) could have been present for members of the Cordilleran *Campanula*. In doing so, we assumed, like others (e.g., Shafer et al. 2010), that the recent refugia were representative of refugia throughout the Pleistocene. This is different from assuming that the glacial cycles were all the same. It is well known that each glacial cycle was different in magnitude and duration from the others (Gibbard et al. 2007). Although the extent of the refugia likely varied from one glacial cycle to the next, the locations probably varied little (especially for those since the mid-Pleistocene transition; the time frame most relevant to speciation in these *Campanula*). Because of this, we felt that our approach provided a foundation for simple biogeographic hypotheses and a starting point for future investigations.

### Niche overlap analysis

We compared the niches of sister taxa to estimate the role of environmental differences in promoting divergence in this group. Principle components analysis (PCA) was used to evaluate the niche space currently occupied by the seven Cordilleran *Campanula* based on climate variables located at georeferenced herbarium samples. CRU climate data were used for Canada and Alaska, while PRISM climate data (1971–2000 normals at 800-m resolution) were used for the western US (PRISM Climate Group, Oregon State University, http://prism.oregonstate.edu, Daly et al. 2008). One herbarium record was used per pixel yielding the following sample sizes: *C. aurita* (44), *C. lasiocarpa* (246), *C. parryi* var*. idahoensis* (24), *C. parryi* var*. parryi* (239), *C. piperi* (41), *C. scabrella* (83), and *C. scouleri* (203).

Using the approach of Broennimann et al. (2012) with R code provided by O. Broennimann, niche overlap was calculated using the *D* metric, which ranges between 0 (no overlap) and 1 (full overlap); randomization tests (100 repetitions) were used to assess niche equivalency and similarity. The null hypothesis for the niche equivalency test is that the two niches being compared are not different; the null hypothesis for the niche similarity test is that the two niches are not more similar than random niches.

### Morphological analyses

We compared vegetative and floral morphology of sister taxa to estimate the role of differing selective pressures imposed by the pollinator communities on divergence in this clade. For pollinators to have mediated speciation, characters associated with floral morphology would best delimit taxa. Morphological data were collected from fresh and dried herbarium specimens using individuals from throughout the species' range in western North America. We compared 28 morphological character traits describing discrete qualitative (Table2) and continuous quantitative (Table3) characters. For the purposes of this study, corolla height and width describe parameters of a flowers silhouette when fully expanded, while corolla length refers to the true length of material from the rim of the hypanthium to the tip of the petal. Data on seed morphology were taken from the work of Shetler and Morin (1986) and supplemented with new measurements from seeds of three individuals of *C. parryi* var*. idahoensis* using scanning electron microscopy.

**Table 2:** Categorical characters for floral, vegetative, and seed morphology.

Taxon	*n*	Flowers/stem	Flower shape	Hypanthium hair type	Pollen color	Upper leaf shape	Upper leaf margin
*Campanula aurita*	10	5	Rotate-subrotate	Glabrous	Purplish	Narrow-elliptic	Inconspicuous-serrate
*Campanula lasiocarpa*	10	2	Tubular-campanulate	Villous	Purple	Narrow-elliptic	Mucronate
*Campanula parryi* var*. idahoensis*	20	2	Tubular-campanulate	Hirtellous	White-yellow	Narrow-elliptic	Inconspicuous-serrate
*Campanula parryi* var*. parryi*	10	2	Broadly campanulate	Glabrous	Yellow	Narrow-elliptic	Sparse-serrate
*Campanula piperi*	40	3	Rotate-subrotate	Hirtellous	Purple	Narrow-elliptic	Mucronate
*Campanula scabrella*	30	3	Broadly campanulate	Hirtellous	Purple	Narrow-elliptic	Entire
*Campanula scouleri*	10	3	Rotate-subrotate	Chaffy	Yellow	Ovate	Conspicuous-serrate
*Campanula aurita*	–	Seed wings	Seed shape long	Seed shape cross-section	Seed surface	Lower leaf shape	Lower leaf margin
*Campanula lasiocarpa*	–	Periphery ridge	Oblong	Narrowly ovate	Interrupted-striate	Oblanceolate	Inconspicuous-serrate
*Campanula parryi* var*. idahoensis*	–	No ridge	Fusiform-ovate	Terete	Striate	Oblanceolate	Mucronate
*Campanula parryi* var*. parryi*	–	One sided ridge	Oblong	Terete	Striate	Oblanceolate	Inconspicuous-serrate
*Campanula piperi*	–	Opposite hilum	Elliptical	Terete	Striate	Oblanceolate	Sparse-serrate
*Campanula scabrella*	–	Ridge on hilum end	Narrowly elliptical	Terete	Striate	Oblanceolate	Mucronate
*Campanula scouleri*	–	One sided ridge	Oblong	Ovate	Striate	Oblanceolate	Entire

Seed data on *C. parryi* var. *idahoensis* were collected herein. Data on seed morphology for all other taxa are from Shetler and Morin (1986).

**Table 3:** Continuous morphological characters.

Taxon	*Campanula aurita*	*Campanul lasiocarpa*	*Campanul parryi* var. *idahoensis*	*Campanul parryi* var. *parryi*	*Campanul piperi*	*Campanul scabrella*	*Campanul scouleri*
*n*	10	10	20	10	40	30	10
Corolla height:width	0.3 (0.1)	1.0 (0.2)	1.0 (0.2)	0.8 (0.1)	0.5 (0.1)	0.6 (0.3)	0.4 (0.2)
Corolla width	22.6 (3.9)	23.3 (4.4)	10.6 (2.7)	19.1 (5.2)	20.8 (3.9)	13.7 (4.4)	9.4 (3.2)
Corolla height	7.9 (1.9)	22.8 (2.2)	10.0 (1.5)	15.1 (1.8)	9.5 (2.3)	7.0 (1.6)	3.8 (0.8)
Corolla length	12.2 (1.7)	25.4 (2.0)	10.6 (1.6)	15.0 (1.8)	12.9 (2.0)	8.7 (1.5)	7.9 (1.4)
Petal lobe length	10.3 (1.8)	9.2 (1.0)	3.9 (0.4)	7.9 (1.7)	9.5 (1.4)	6.0 (1.4)	4.4 (1.2)
Fused corolla length	2.1 (0.3)	16.2 (1.5)	6.8 (1.3)	7.6 (1.1)	3.4 (0.9)	2.7 (0.8)	3.5 (0.6)
Style beyond corolla	2.6 (1.1)	−8.2 (1.7)	−1.7 (1.2)	−6.5 (1.6)	1.3 (2.6)	0.2 (1.5)	8.6 (1.2)
Style:stigma length	11.0 (1.6)	13.5 (1.7)	8.2 (1.2)	8.7 (1.8)	8.8 (1.6)	6.5 (1.4)	12.8 (1.0)
Sepal length	5.2 (1.4)	8.5 (1.2)	3.8 (1.9)	10.5 (3.2)	5.3 (1.2)	3.6 (1.0)	4.7 (1.8)
Petal base width	2.7 (0.5)	8.1 (0.9)	3.7 (0.8)	5.4 (1.9)	5.6 (1.2)	3.0 (1.6)	2.2 (0.7)
Hypanthium length	4.4 (0.9)	4.7 (0.9)	3.6 (1.0)	5.3 (0.9)	3.4 (0.7)	3.1 (0.8)	3.1 (0.9)
Hypanthium length:width	1.1 (0.2)	1.0 (0.1)	1.4 (0.3)	1.5 (0.3)	0.8 (0.1)	1.2 (0.2)	1.1 (0.3)
Stem length	163.0 (40.3)	46.1 (20.3)	117.5 (39.7)	160.0 (53.1)	30.1 (13.8)	38.4 (18.3)	187.5 (89.8)
Hypanthium width	4.1 (0.5)	4.7 (1.0)	2.7 (0.4)	3.7 (0.7)	4.2 (0.7)	2.6 (0.6)	2.8 (0.4)
Seed length	1.2	0.7	0.9	0.6	0.7	0.85	1.0
Seed length:width	2.0	2.0	2.6	1.8	2.0	2.4	2.0

Sample grand means are given with standard deviation in parenthesis. Seed length and length/width ratio, with exception of *C. parryi* var. *idahoensis*, adapted from Shetler and Morin (1986); standard deviation not provided. Measurements are given in mm.

Classification trees (De'ath and Fabricius 2000) were used to determine the number of different taxonomic groups in the Cordilleran *Campanula* samples and to estimate which of the categorical variables (Table2) were important in differentiating among taxa. Analyses were conducted in R using package TREE (Ripley 2012).

We also employed discriminant function analysis in R (package MASS; Venables and Ripley 2002) to determine the relative strength with which the continuous morphological characters (Table3) contributed to the correct classification of the taxonomic groups. Prior to running the discriminant analysis, data were assessed for normality using quantile–quantile plots for each character. To approximate normal distributions, characters whose distributions departed from normality were log-transformed.

## Results

### Paleodistribution modeling

**Figure 3:**
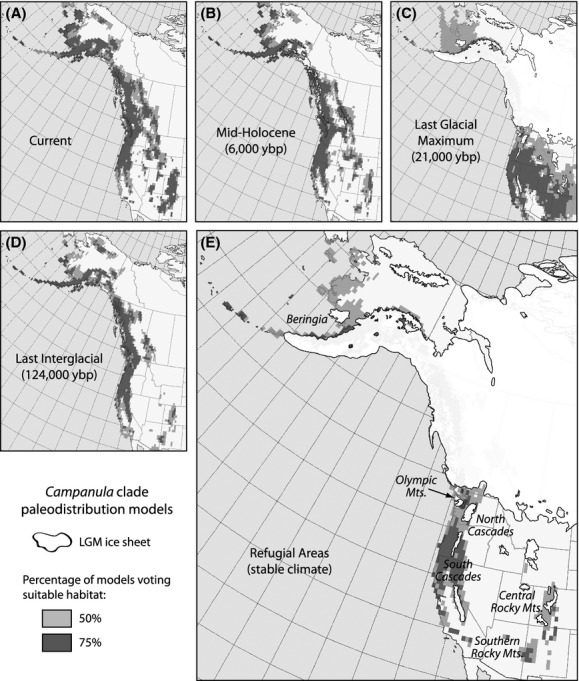
Paleodistribution models and hypothetical refugia for the Cordilleran *Campanula*. Maps were generated for four time periods: (A) current, (B) the mid-Holocene, (C) the Last Glacial Maximum (LGM), and (D) the Last Interglacial. Areas of stable climate over time are indicated in the (E) refugial map. The white represents the extent of LGM ice. Pixels with half of the ecological niche models voting suitable habitat are in light gray; those with 75% of the models voting suitable habitat are in dark gray.

All models met the threshold for inclusion in the ensemble, with all AUC scores >0.8, indicating very good model performance. The inferences for the present time period showed potential mixing between Beringia and the southern mountains, but isolation among southern refugia (Fig.3A). For the mid-Holocene, a Beringia/southern division is weak and there was a strong signal of isolation among southern refugia (Fig.3B). At the LGM, Beringia was isolated from mountains south of the Ice Sheets, but there were ample connections within regions (Fig.3C). During the LIG, suitable habitat was separated between the west and east, with drastic isolation of the Southern and Northern Rocky Mountains (Fig.3D). By combining the results for all time periods, we identified six potential refugia (Beringia, the Olympic Mountains, the North and South Cascades, and the Central and the Southern Rocky Mountains; Fig.3E), which were used to develop the three biogeographic hypotheses: isolation between the aforementioned multiple refugia (MR), northward expansion (NE) from the southern mountain refugia, and southward expansion (SE) out of the north.

### Molecular analyses and biogeographic hypotheses

Removing recombinants left a total of 181 nDNA sequences (across 10 new ALPS loci) for the Cordilleran *Campanula* and three related species. Tests of neutrality on these and the ITS data (reported in Wendling et al. 2011) detected a signature of population growth or selection for three loci: ALPS 04, 06, and 19 (Table1). The models of DNA substitution inferred from JMODELTEST and the relative rates gleaned from *BEAST (Table1) show the variation inherent in the stochastic nature of the evolutionary process for noncoding loci.

**Figure 4:**
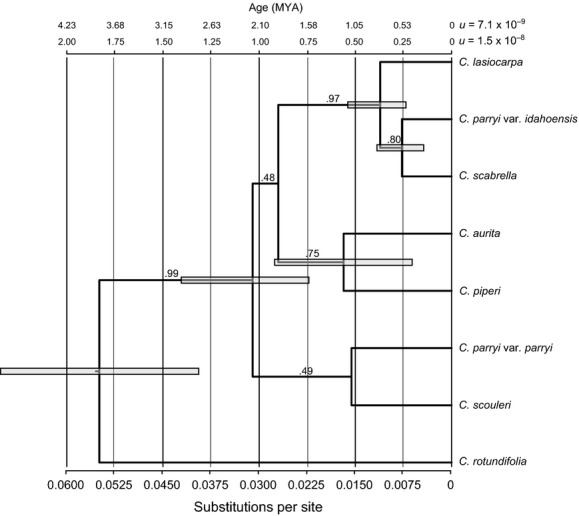
Phylogeny and divergence times for the Cordilleran *Campanula* based on the *BEAST analysis of eleven nuclear loci. Numbers above the branches represent Bayesian posterior probabilities for the node. The timescales at the top of the figure indicate divergence times as a function of substitutions per site (at the bottom) for the fast and slow mutation rates. Gray bars show the 95% highest posterior density for age estimates.

Several important findings concerning the evolutionary history of the Cordilleran *Campanula* emerged from the *BEAST phylogenetic analyses of nDNA (Fig.4). First, *C. rotundifolia* and the Cordilleran *Campanula* split approximately 1967 (range 1462–2925, given the error in the faster mutation rate [±0.5 × 10^−8^]) to 4154 (3750–4570, for the slow mutation rate [±0.7 × 10^−9^]) KYA using the faster and slower mutation rates, respectively. Concerning the Cordilleran Clade itself, (1) the seven taxa form a monophyletic group (posterior probability [pp] = 0.99) that began to diverge about 1030 (775–1550) to 2183 (1987–2384) KYA, (2) the two varieties of *C. parryi* (var. *parryi* and var. *idahoensis*) are indeed separate species as earlier analyses suggested (Wendling et al. 2011), and (3) a mid-Pleistocene radiation occurred about 560 (420–840) to 1183 (1076–1312) KYA for *C. piperi* and *C. aurita* (pp = 0.48), 510 (382–765) to 1077 (980–1195) KYA for *C. scouleri* and *C. parryi* var. *parryi* (pp = 0.49), and more recently at approximately 386 (290–580) to 816(743–906) KYA for *C. lasiocarpa*, *C. scabrella,* and *C. parryi* var. *idahoensis* (pp = 0.97) with a final speciation event splitting *C. scabrella* and *C. parryi* var. *idahoensis* (pp = 0.80) at approximately 240 (180–360) to 507 (461–562) KYA.

**Figure 5:**
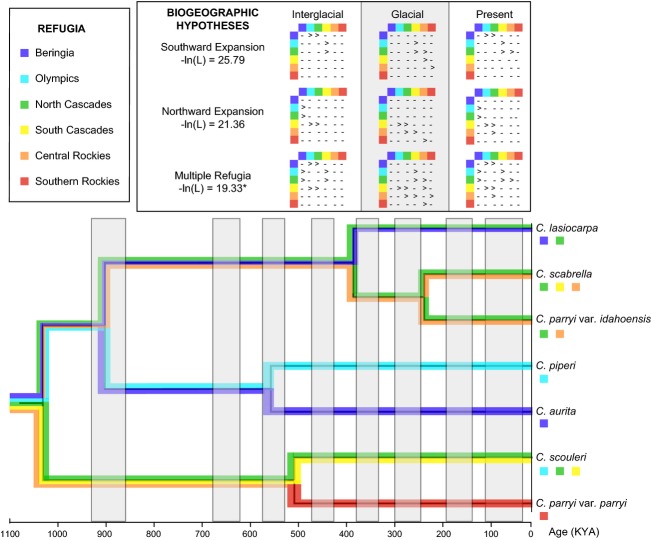
Most likely reconstruction of geographic range dynamics for the multiple refugia hypothesis with root age 1030 KYA. Only the chronology for the faster rate is shown for simplicity. For all biogeographic hypotheses, potential dispersal was inferred from the paleodistribution models (Fig.3) with 0–14 KYA based on the present, earlier interglacials based on LIG, and glacial periods from the LGM. White bars represent interglacial periods and gray bars denote glacials. Dispersal matrices are shown for each biogeographic hypothesis (“>” for directional dispersal and “-” for no dispersal) with an * denoting the most likely hypothesis – multiple refugia. Here, the phylogeny from Figure4 was pruned to the Cordilleran *Campanula*.

The multiple refugia hypothesis was the most likely scenario for both mutation rates (−ln(*L*) = 19.33, 19.66; Table4) to have given rise to the tree generated from the multi-locus dataset in *BEAST (Fig.5). The other two hypotheses were at least two log-likelihood units less likely. Thus, our analyses support divergence among refugia. The ancestral area for the clade remains uncertain, potentially some combination among all refugia (Table4).

**Table 4:** Outcomes of biogeographic hypothesis testing in LAGRANGE.

Root age (KYA)	Hypothesis	−ln(*L*)	Dispersal (per year)	Extinction (per year)	Ancestral Refugium	−ln(*L*)
1030^1^	Multiple refugia	19.33*	0.0046	0.0010	N Cascades	21.29
					Beringia	22.23
					Beringia/N Cascades	22.24
					N Cascades/C Rockies	22.31
					Olympics	22.64
					S Cascades	22.65
					N Cascades/S Cascades	23.07
					S Cascades/Olympics	23.21
					N Cascades/Olympics	23.23
1030^1^	Northward expansion	21.36	0.0130	0.0012	C Rockies/S Rockies	22.46
					S Rockies	22.94
1030^1^	Southward expansion	25.79	0.0057	0.0006	N Cascades/Beringia	27.10
					Beringia	27.43
					Beringia/Olympics	28.25
2183^2^	Multiple refugia	19.66*	0.0025	0.0006	N Cascades	21.71
					N Cascades/C Rockies	22.59
					S Cascades	22.77
					Beringia	22.79
					Beringia/N Cascades	22.93
					Olympics	22.96
					C Rockies	23.29
					N Cascades/S Cascades	23.49
2183^2^	Northward expansion	20.98	0.0064	0.0006	C Rockies/S Rockies	21.99
					S Rockies	22.76
2183^2^	Southward expansion	23.99	0.0031	0.0004	Beringia/N Cascades	25.14
					Beringia	25.51

Ancestral areas inferred for the root node of each model within two log-likelihood units of the most likely area are shown. The root ages were estimated using

1 *u* = 1.5 × 10^−8^ and

2 *u* = 7.1 × 10^−9^. The most likely scenario is shown with an * for each mutation rate.

### Niche overlap analysis

**Figure 6:**
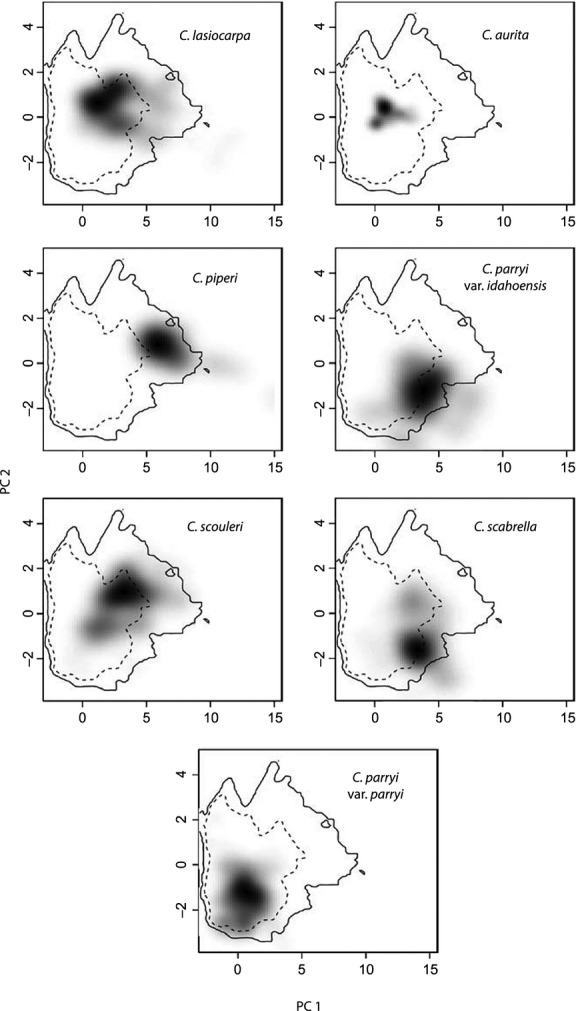
Plots of niche space for the Cordilleran *Campanula*. Shading indicates the density of the PC scores for occurrence records in each clade. The contour lines indicate 50% (dotted) and 100% (solid) of the available environment. In some cases, shading extends outside the available environment due to kernel smoothing (described in Broennimann et al. 2012).

Plots of niche space (Fig.6) illustrated environmental differences and similarities among taxa. Principle component (PC) axes 1 and 2 explained 49% and 25% of the variance (Table S4), respectively, with precipitation of the wettest and driest months loading most strongly onto PC1 (positively) and annual temperature range loading less strongly (negatively). Summer minimum temperature loaded most strongly onto PC 2 (positively) with summer mean diurnal range loading less strongly (negatively). The plots of niche space illustrate the influence of environment on speciation events. For example, *C. piperi* shows the most positive values on PC1, reflecting its distribution in the relatively wet and temperate Olympic Peninsula, while PC1 values are near zero for its sister species, *C. aurita*, reflecting its more interior distribution. The same pattern can be seen for divergence between *C. scouleri* and *C. parryi* var. *parryi*. Likewise, in both comparisons, the species tend to sort on PC2 reflecting lower or higher summer mean diurnal ranges based on coastal or interior climates, respectively. In contrast, *C. scabrella* and *C. parryi* var. *idahoensis* cannot be distinguished based on niche space.

Furthermore, in all comparisons but one, that of *C. aurita* and *C. parryi* var. *parryi* (both interior species), tests of niche equivalency rejected the null hypothesis that the niches were not different (*P* = 0.0198). Other tests of niche similarity were mixed (Table5), so here we focus on subclade comparisons to assess the relative role of differing environmental pressures on divergence. While the two subclades comprised of *C. piperi-C. aurita* and *C. scouleri-C. parryi* var. *parryi* exhibited different niches based on both equivalency and similarity, the niche overlap for *C. lasiocarpa-C. scabrella-C. parryi* var. *idahoensis* was equivalent and similar. This is not surprising as the latter taxa are found in close proximity in the field and occupy similar environmental conditions (Fig.6). It should also be noted that the widely distributed *C. lasiocarpa* shared niche similarity with four of the six other species.

**Table 5:** Results (*P*-values) of niche similarity tests, where the null hypothesis is that two niches are not more similar than random niches.

	*Campanula aurita*	*Campanul lasiocarpa*	*Campanul parryi* var. *idahoensis*	*Campanul parryi* var. *parryi*	*Campanul piperi*	*Campanul scabrella*	*Campanul scouleri*
*C. aurita*	–	0.7327	0.5545	0.5149	0.7921	0.7525	0.6931
*C. lasiocarpa*	0.2970	–	**0.0396**	0.0792	**0.0198**	**0.0198**	**0.0198**
*C. parryi* var. *idahoensis*	0.3366	**0.0198**	–	0.2772	0.1584	**0.0198**	0.3762
*C. parryi* var. *parryi*	0.9703	0.6535	0.3762	–	**0.0396**	0.1584	0.1188
*C. piperi*	0.2178	**0.0396**	**0.0396**	0.3366	–	0.0594	**0.0198**
*C. scabrella*	0.4158	**0.0198**	**0.0198**	0.3960	0.0990	–	0.3564
*C. scouleri*	0.2574	**0.0198**	0.0792	0.0594	**0.0198**	0.1782	–

The top right portion of the table shows results comparing row taxa to column taxa; bottom left are comparisons of columns to rows. Significant *P*-values (<0.05) are in bold.

### Morphological analyses

Based on the combination of all the categorical data, the classification tree analysis separated the individuals into the seven taxonomic groups without error when using the combined dataset or only the flower or seed datasets. A classification tree based on the vegetative characters only uncovered five taxonomic groups (misclassification error rate = 0.16), with *C. lasiocarpa* and *C. piperi* grouping together and *C. aurita* and *C. parryi* var. *idahoensis* forming another group because of their exact overlap in vegetative traits (Table2).

**Figure 7:**
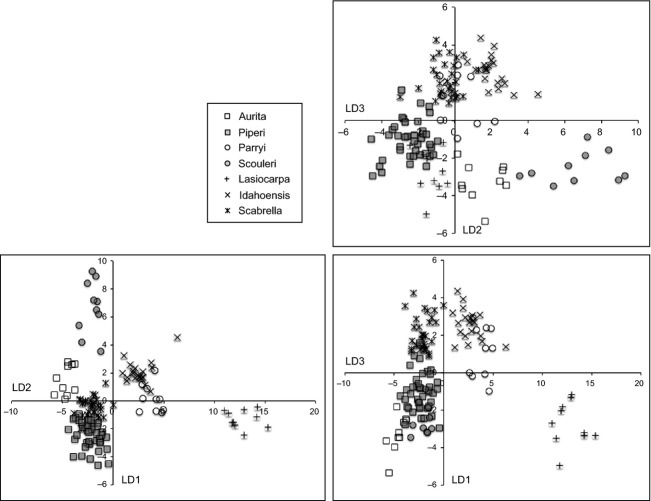
Discriminant analysis of the continuous floral characters. Comparisons between the first through third discriminant functions are given. The taxa are abbreviated by their specific or varietal epithet: aurita (*Campanula aurita*), idahoensis (*Campanula parryi* var. *idahoensis*), lasiocarpa (*Campanula lasiocarpa*), parryi (*Campanula parryi* var. *parryi*), piperi (*Campanula piperi*), scabrella (*Campanula scabrella*), and scouleri (*Campanula scouleri*).

In the discriminant function analysis, 83.8% of the variance was explained by three coefficients of linear discriminants (LD) (Table6). The first function (LD1) characterized the groups based mostly on the mean corolla length. Both the corolla height to width ratio and the hypanthium length to width ratio contributed to LD2, while the hypanthium length to width ratio again was important in LD3. Fused corolla length and sepal length also contributed to the less important linear discriminants. The distinctions among all taxa, and importantly between *C. parryi* var. *parryi* and *C. parryi* var. *idahoensis*, are clear in the pairwise plots of the discriminant functions (Fig.7). Within each of the three subclades, floral morphology clearly delineated species. *C. piperi-C. aurita* LD2 and LD3 were important underscoring differences in the hypanthium. *Campanula scouleri-C. parryi* var. *parryi* were strongly differentiated by all three LDs. Finally, *C. lasiocarpa-C. scabrella-C. parryi* var. *idahoensis* were all quite different but with some overlap between *C. scabrella-C. parryi* var. *idahoensis* in LD3.

**Table 6:** Coefficients of linear discriminants for the continuous floral characters.

	LD1	LD2	LD3
% Variance explained	54.8	16.4	12.6
Character			
Log mean corolla length (mm)	**6.711**	−1.706	−1.964
Corolla height:width	0.453	−**2.631**	1.326
Log hypanthium length:width	−1.047	**2.502**	**3.647**
Log corolla height (mm)	−0.013	−1.308	−0.067
Log fused corolla length (mm)	0.603	1.109	0.988
Log mean sepal length (mm)	0.263	−0.853	−0.862
Corolla width (mm)	0.055	−0.150	0.017
Mean petal lobe length (mm)	−0.955	0.023	−0.089
Mean petal lobe width at base (mm)	0.042	−0.112	0.132
Distance style clears corolla (mm)	−0.174	0.065	−0.081
Style–stigma length (mm)	0.081	0.306	−0.307
Hypanthium length (mm)	0.104	−0.505	0.042
Hypanthium width (mm)	−0.784	0.500	−0.084
Log stem length (mm)	0.982	1.072	−0.583

The largest absolute correlations between any variable and discriminant functions are indicated in bold.

## Discussion

The comparative phylogeographic study of plants in the Pacific Northwest by Soltis et al. (1997) hypothesized that the present-day distribution of plants was molded by isolation in one or multiple refugia during Pleistocene glaciations and that genetic differentiation could have arisen through drift or in response to differing selection pressures of the environment. Many recent studies (reviewed in Shafer et al. 2010) support a multiple refugia hypothesis for the region. *Oxyria digyna* (L.) Hill (Allen et al. 2012), for example, not only provided support for multiple refugia, but also the possibility of cryptic refugia in north British Columbia. Likewise, the persistence of *Orthilia secunda* (L.) House in multiple western refugia led to the emergence of endemic haplotypes (Beatty and Provan 2010). Our findings follow up on these, strongly supporting the hypothesis and going further to suggest that isolation between multiple refugia, along with the differing biotic and abiotic selection pressures, promoted speciation and the rise of several endemic *Campanula* species.

The Cordilleran Clade of *Campanula* arose in the early Pleistocene and radiated rapidly across the mountains of western North America. We inferred that the Cordilleran *Campanula* arose approximately 1–2 MYA and that subsequent speciation within the clade (at about 0.5–1 MYA) was associated with geographic isolation between multiple refugia – Beringia, the Olympics, the North and South Cascades, and the Central and Southern Rockies. The diversity and high degree of endemism exhibited by this clade is likely the combined effect of climate oscillations, the rugged alpine habitat, heterogeneous environments, and a proclivity among the *Campanula* for changes in floral morphology. The shifting climate at times separated populations north and south of the Great Ice Sheets and continually forced them to track suitable habitat. Most of the Cordilleran *Campanula* inhabit the arctic-alpine tundra, a fact that served to further isolate populations scattered across mountaintops during warm interglacials, resulting in a patchwork of diverging taxa. Each of those geographic regions differs environmentally, owing to the great latitudinal expanse of the cordillera in North America and varying distance from the coast. Not only has the abiotic environment differed among locales, but the selective pressures imposed by the pollinator community probably varied as well. These factors, along with the tendency for changes in floral morphology, likely gave rise to the genetic and morphological diversity observed in this clade.

The evaluation of such biogeographic scenarios relies on using contemporary data to estimate divergence times. Several factors could have negatively impacted our ability to accurately estimated divergent dates. First, in some instances, genetic sampling was limited to a single individual, which can affect branch length estimates via node density effects. Because several population models yielded the same tree topology and branch lengths, our sampling scheme probably did not bias the phylogenetic inferences. Yet, the question of monophyly within species remains open, requiring a population-level study. Moreover, incomplete lineage sorting is likely given the inferred recent time frame, which could produce misleading phylogenies for taxa with small sample sizes (Maddison and Knowles 2006). We attempted to overcome this issue by sequencing multiple nuclear loci. Second, because fossils were not available to calibrate the branch lengths on our phylogeny, we used a range of mutation rates (as per Saquet 2013) for plant autosomal loci to estimate divergence times. By adopting “slow” and “fast” rates, we hoped to cover the range of potential divergence times. Furthermore, those rates were based on “per generation,” and we estimated the generation time of our focal taxa to be 2 years (based on that of *C. rotundifolia*). This is realistic for these arctic-alpine perennials, but underestimating generation time would overestimate divergence times. Given those caveats, our time estimates should be viewed with caution. Regardless of the time frame, both the “slow” and “fast” rates supported the multiple refugia hypothesis, bolstering that biogeographic inference.

Though the exact time frame remains in question, the paleodistribution models, multi-locus phylogeny, and biogeographic analyses all support a rapid radiation of the Cordilleran Clade that was driven by the geographic isolation across the mountains of western North America imposed by the Quaternary climate cycles. Along with other arctic-alpine plants (Brochmann and Brysting 2008), the clade arose about 1–2 MYA and diversified in bursts across refugia (multiple refugia hypothesis) (Fig.5). Most of the speciation events in the Cordilleran *Campanula* occurred directly after the middle Pleistocene transition (MPT; from 1250 to 700 KYA; Shackleton and Opdyke 1976; Pisias and Moore 1981; Clark et al. 2006), which marked the beginning of the more severe, 100 KY glacial cycles. This time frame also fits with those inferred for the divergence of other Campanulaceae (Roquet et al. 2009; Mansion et al. 2012), illustrating the importance of this period for speciation within this genus and family. Moreover, this climatic transition has been linked to divergence of diverse arctic-alpine plants from Crassulaceae in western North America (DeChaine and Martin 2005; DeChaine et al. 2013a,b) to Primulaceae in the Maritime Alps (Casazza et al. 2013) and thus likely had a profound impact on the evolution of the flora. Finally, the only widespread Beringian species, *C. lasiocarpa*, probably recolonized Eurasia in the late Pleistocene, yielding the current geographic range of the clade.

How the Quaternary climate cycles impacted the degree of refugial isolation differed depending on geographic location: glacials connected southern populations but isolated the north, while interglacials fragmented the south and united the north. In essence, the two regions have been out of sync. During glacial periods, the Laurentide and Cordilleran ice sheets divided the landscape latitudinally (Richmond 1965), with suitable habitat remaining only in mountain refugia south of the Ice Sheets and, to a lesser degree, Beringia to the north (Fig.3E). The opposite is true for the distribution of available habitat during the warm periods (Figs.3A,B,D), wherein dispersal became possible between the north and south, but the south experienced fragmentation with the Rockies becoming isolated from the coastal ranges. Apparently, the more pronounced fragmentation throughout the south during warm periods was instrumental in the process of divergence and the derivation of numerous endemics in this group and probably other alpine plants. Yet, the ample connections among southern regions during glacial periods would have enhanced opportunities for gene flow among populations (Fig.3C), suggesting that factors beyond just geographic isolation generated and/or upheld species boundaries.

Not only have taxa been isolated throughout the Quaternary, due to the shifting climate and dramatic topography, but these features in combination created environmental differences across the expansive latitudinal range and coastal-continental breadth occupied by the Cordilleran *Campanula*, likely fostering further genetic divergence through varying selection pressures (Givnish 2010). Broadly speaking, sister taxa tend to occupy niches that are similar, but not equivalent. Many comparisons meet the above expectation, including most comparisons with the widely distributed *C. lasiocarpa*. However, because the Cordilleran *Campanula* are so broadly and patchily distributed across a range of climatic conditions, this generalization does not stand for all comparisons. Unsurprisingly, the taxa sort on continental-scale gradients (e.g., coastal vs. interior climates) driven by precipitation (PC 1) and temperature (PC 2) variables. But, the influence of the environment on individual divergence events varied. For example, the subclades of *C. piperi-C. aurita* and *C. scouleri-C. parryi* var. *parryi* illustrated the important role of abiotic differences between species. In both subclades, the disjunct distributions of sister taxa occur at either end of the coastal-interior gradient, underscoring the importance of both geography and environmental conditions for those species. But, the other subclade, *C. lasiocarpa-C. scabrella-C. parryi* var. *idahoensis,* showed both niche similarity and equivalency, suggesting little if any influence of environment on divergence. Thus, in some cases, differing abiotic conditions may have promoted character displacement and further cemented the taxonomic boundaries initiated in allopatry, but that was not the case for all speciation events.

Reproductive characters associated with flower and seed morphology provide the final layer to understanding divergence within the Cordilleran *Campanula*. The vegetative characters that we examined were not good predictors of species identity, and likely exhibited plastic variation in response to broad environmental conditions (Eddie and Ingrouille 1999; Roquet et al. 2008). In contrast, petal length and degree of fusion (campanuliform) were among the most important factors in discriminating taxa based on morphology. While the relative importance of different characters varied between the subclades (Table6, Fig.7), in each case floral morphology was a key to distinguishing species. Flower shape, particularly rotate versus campanulate, has been used in *Campanula* as a proxy for generalist versus specialist pollinators, respectively, and may be related to selective pressures imposed by the local pollinator community (Roquet et al. 2008). Roquet et al. (2008) hypothesized that attracting a more general suite of pollinators might be advantageous in a less predictable climate, such as that of the Pleistocene, a hypothesis that fits the appearance of the rotate shape in this clade (*C. scouleri* and *C. piperi-C. aurita*). If those lineages that retained the campanulate shape (*C. parryi* var*. idahoensis, C. lasiocarpa, C. parryi* var*. parryi,* and *C. scabrella*) attracted specific pollinators, then floral form might have strengthened genetic divergence associated with refugial isolation.

Our analyses of the Cordilleran *Campanula* have uncovered important clues to understanding the process of speciation in a system that has experienced repeated and protracted periods of potential gene flow. At least for populations inhabiting the southern mountains, cool glacial periods provided increased opportunities for population mixing. But, our phylogenetic and morphological analyses supported a scenario of only rare instances of panmixia for arctic-alpines in western North America. We attempted to resolve this potential conflict by including the influences of both the abiotic and biotic environment in our analyses. Other plants in shifting environments have undergone speciation in the face of ongoing gene flow, such as *Heliotropium* in the Atacama Desert (Luebert et al. 2013). Indeed, as in the case of *Heliotropium,* pollinator-mediated reinforcement could have been a major factor in the divergence of the Cordilleran *Campanula*. For angiosperms, reinforcement works most strongly when reproductive isolation is enhanced by changes in floral morphology and acts only when species have the opportunity to hybridize (Whalen 1978; reviewed in Hopkins 2013). While reinforcement could help to explain our findings, the taxonomic differences could also be attributed to a simpler model of character displacement. The Cordilleran *Campanula* and other arctic-alpines provide excellent opportunities to further study this problem.

## Conclusions

We inferred the response of the Cordilleran *Campanula* to the Quaternary glacial cycles by integrating three independent estimates of their evolutionary history – ecological niche models, multi-locus genetic sequence data, and morphological variation. Each of these approaches has its idiosyncratic strengths, but when combined, much more of the evolutionary history of the group is made evident. The ecological niche models provide the necessary geographic component for developing hypotheses that are ultimately linked to allopatric divergence. Phylogenetic analyses of multi-locus genetic data are an excellent means of estimating relationships and the timing of divergence among taxa and testing models of divergence. And finally, the statistical tests of morphological differences place the divergence in the context of phenotype, which in this case was likely driven by a combination of differing environmental and pollination pressures. These integrated datasets yielded a more comprehensive estimate of the factors underlying divergence in this clade on the whole and the relative importance on individual speciation events than would have been possible by any one of them singularly – revealing the timing and potential environmental and biotic factors associated with the radiation in this group. The consequence of the Quaternary for the Cordilleran *Campanula* has been rapid diversification that in the end yielded numerous narrowly endemic species. Each portion of the range for this clade, especially those regions identified as refugia, is critical to conserving species and the processes that have given rise to the diversity of alpine plants in North America.

Our findings highlighted the way in which the unique topography and climatic history of the western North American landscape – mountain chains spanning an immense latitudinal range and at times occurring as disjunct mountain blocks – influenced the evolutionary history of arctic-alpine plants. As much as these *Campanula* are representative of the arctic-alpine flora, our study suggests that a combination of repeated rounds of geographic isolation, environmental differences among regions, and biotic pressures promoted divergence of lineages, speciation, and the generation of geographically narrow endemics. These factors, along with others such as polyploidization and hybridization, have permitted and perhaps encouraged speciation of plants through the Quaternary.

## Data Accessibility

GenBank accessions: KM279093-KM279276. Final DNA sequence assembly: The sequence alignments and phylogenies have been archived in TREEBASE, http://purl.org/phylo/treebase/phylows/study/TB2:S16226

## Supporting Information

**Table S1.** Herbarium accession and locality data for geographic and genetic* samples.

**Table S2.** Locality and character data for morphological samples.

**Table S3.** Molecular data matrix for number of samples per *Campanula* taxa across loci. **Table S4.** Variable loadings and correlations with the first two PC axes of the niche overlap analysis.
