# Supplementary material for: Integrating environmental, molecular, and morphological data to unravel an ice-age radiation of arctic-alpine Campanula in western North America
Source: Ecol Evol. 2014 Sep 26;4(20):3940–59. doi: 10.1002/ece3.1168 (PMC4242577; doi:10.1002/ece3.1168)
Supplement: Supplementary file 3 [file ece30004-3940-SD3.docx]

Supporting Information

Supplemental Table 1. Herbarium accession and locality data for geographic and genetic* samples.

Supplemental Table 2. Locality and character data for morphological samples.

Supplemental Table 3. Molecular data matrix for number of samples per *Campanula* taxa across loci.

|  | n | ALP03 | ALP04 | ALP06 | ALP13 | ALP14 | ALP19 | ALP24 | ALP25 | ALP31 | ALP32 | ITS |
| --- | --- | --- | --- | --- | --- | --- | --- | --- | --- | --- | --- | --- |
| *C. aurita* | 1 | 1 | 1 | 1 | 1 | 2 | 1 | 1 | 1 | 1 | 2 | 1 |
| *C. lasiocarpa* | 7 | 7 | 8 | 5 | 5 | 6 | 6 | 3 | 1 | 5 | 2 | 1 |
| *C. parryi* var*. idahoensis* | 1 | 1 | 2 | 1 | 1 | 2 | 1 | 1 | 1 | 1 | 1 | 1 |
| *C. parryi* var*. parryi* | 1 | 1 | 2 | 1 | 1 | 2 | 2 | 1 | 1 | 1 | 1 | 1 |
| *C. piperi* | 4 | 4 | 3 | 4 | 2 | 6 | 8 | 3 | 3 | 6 | 3 | 1 |
| *C. scabrella* | 4 | 4 | 3 | 4 | 4 | 3 | 4 | 2 | 4 | 3 | 3 | 1 |
| *C. scouleri* | 1 | 1 | 0 | 1 | 1 | 2 | 2 | 1 | 1 | 1 | 1 | 1 |
| *C. rotundifolia* | 1 | 1 | 0 | 1 | 1 | 0 | 1 | 1 | 1 | 1 | 1 | 1 |
| Total: |  | 20 | 19 | 18 | 16 | 23 | 26 | 13 | 13 | 19 | 14 | 8 |

Instances in which the number of samples per taxon for a given locus is greater than the number of individuals per taxon are due to heterozygous individuals.

Supplemental Table 4. Variable loadings and correlations with the first two PC axes of the niche overlap analysis.

| Variable | Loadings | | Correlations | |
| --- | --- | --- | --- | --- |
|  | PC1 | PC2 | PC1 | PC2 |
| annual temperature range | -0.479 | -0.223 | -0.750 | -0.249 |
| mean diurnal range, summer | -0.426 | -0.483 | -0.667 | -0.539 |
| summer minimum temperature | -0.261 | 0.759 | -0.409 | 0.848 |
| precipitation of the driest month | 0.487 | -0.359 | 0.761 | -0.400 |
| precipitation of the wettest month | 0.533 | 0.113 | 0.833 | 0.126 |

Axis 1 explained 49% of the variance, while axis 2 explained 25%.
